# Systematic Analysis of MRTFB Phosphoproteomic Datasets Reveals Co-Regulated Kinase Networks and Co-Occurring Phosphosites Associated with the Actin Cytoskeleton Pathway

**DOI:** 10.3390/cimb48090910

**Published:** 2026-09-05

**Authors:** Jetna John, Vaishnavi Gopalakrishnan, Suhail Subair, Athira Perunelly Gopalakrishnan, Akhina Palollathil, Rajesh Raju

**Affiliations:** Centre for Integrative Omics Data Science (CIODS), Yenepoya (Deemed to be) University, Mangalore 575018, Karnataka, India; jetnajohn.study@gmail.com (J.J.); gvaishutiruppur@gmail.com (V.G.); suhailrejeena007@gmail.com (S.S.); athirajrf@yenepoya.edu.in (A.P.G.)

**Keywords:** MRTFB, phosphorylation, actin cytoskeleton, conservation, co-occurrence

## Abstract

**Background:** Myocardin-related transcription factor B (MRTFB) acts as a transcriptional coactivator for serum response factor (SRF) and regulates actin cytoskeletal dynamics. Despite its biological significance, the phosphoregulatory network, upstream kinases, and interactors remain poorly understood. **Methods:** To explore this, we analyzed global phosphoproteomic datasets to identify predominant phosphosites. We categorized phosphosites in other proteins as positively or negatively co-regulated with the predominant phosphosites of MRTFB and also integrated predicted upstream kinases and interactors of MRTFB. Further, phosphosite conservation, co-occurrence patterns, and functional and pathway enrichment analyses of co-regulated proteins were also performed. **Results:** We identified S66 and S921 as predominant phosphosites in MRTFB, with the highest detection frequency across multiple experimental conditions. These phosphosites were found to be conserved within the MRTF family and across multiple species. From the predicted upstream kinases, MAPK1, MAPK3, MAST3, and CSNK1A1 were identified as predicted upstream kinases. Co-occurrence analysis revealed high positive co-occurrence between the predominant phosphosites, S66 and S921, suggesting similar functional roles. Functional enrichment analysis highlighted the involvement of co-regulated proteins in the actin cytoskeleton pathway. **Conclusions:** This study provides the detailed phosphoproteomic landscape of MRTFB, mapping its upstream kinases and co-regulated proteins involved in actin cytoskeleton regulation.

## 1. Introduction

Protein phosphorylation is one of the most studied types of post-translational modifications (PTMs) governing eukaryotic cellular signal transduction [1,2]. Phosphorylation cascades, regulated by distinct kinase families, govern a wide spectrum of cellular functions, making them crucial regulators of signaling fidelity and candidate targets for therapeutic intervention [2]. Reversible phosphorylation can alter the function of a protein, including activity changes, subcellular localization, and protein-protein interactions [3]. Over the past two decades, high-throughput quantitative phosphoproteomics using mass spectrometry coupled with advanced phosphopeptide enrichment techniques, such as IMAC and MOAC, has revolutionized our capacity to capture global phosphodynamics at single-amino-acid resolution [1,4]. However, translating these vast phosphoproteomic datasets into functional signaling networks and identifying functional roles of proteoforms remains a major challenge [1,5]. Identifying predominant phosphosites and their co-regulated upstream kinases is critical for deciphering how key transcriptional coactivators are regulated within upstream signaling networks [5].

Myocardin-related transcription factor B (MRTFB), also known as Megakaryoblastic Leukemia 2 (MKL2), was first identified by Selvaraj and Prywes in 2003, by searching genomic databases for sequences similar to myocardin and MKL1. The full-length MKL2 coding sequence was cloned using the RT-PCR method [6]. MRTFB is a transcription coactivator of serum response factor (SRF) [7], a ubiquitous MADS-box transcription factor that regulates expression of genes related to muscle differentiation, cell growth, and cytoskeletal organization by binding to CArG box DNA elements [8]. The protein MRTFB belongs to the MRTF family, which also includes myocardin (MYOCD) and MRTF-A (MKL1, MAL). The structural hallmark of this family is the presence of evolutionarily conserved domains that facilitate homo- and heterodimerization with other family members, as well as interactions with actin binding and transcriptional activation [9]. The presence of a long 3′ untranslated region (UTR) is a unique characteristic that distinguishes MRTFB from other family members [6]. The *MRTFB* gene is located on chromosome 16p13.12 and comprises 17 exons spanning more than 126 kb of genomic DNA [6,7]. The protein encoded by *MRTFB* consists of 1088 amino acids, has an approximate molecular weight of 118.1 kDa, and shares 44% and 36% sequence identity with MKL1 and myocardin, respectively [6,10,11]. The domains of the MRTF family are highly conserved, including an N-terminal MKL homology domain containing three RPEL motifs (rich in Arg-Pro-Glu-Leu) that are responsible for G-actin binding; a basic region (Arg/Lys-rich) situated adjacent to the RPEL regions is crucial for nuclear localization; the glutamine-rich region (Q-rich), which is involved in mediating transcriptional activity; the SAP domain, which is a DNA-binding motif involved in SRF-independent transcriptional regulation; and the leucine zipper-like domain (LZ) located at the C-terminal region, which facilitates the homo- or hetero-dimerization of the protein [6,12]. While expressed ubiquitously, the MRTFB shows abundance in brain tissue (RPKM 11.0) and testis (RPKM 5.1), and also in 24 other tissues [13].

MRTFB functions as a transcriptional co-activator involved in smooth and skeletal muscle differentiation, cardiovascular development, and regulation of synaptic morphology [14,15,16]. Similar to other family members, MRTFB binds to and activates SRF at CArG box-regulated promoters; however, this activation is specific to the SRE (serum response element) site [6]. Through this partnership with SRF, MRTFB regulates the expression of specific tumor suppressor genes such as *MCAM* and *SPDL1*, thereby regulating growth and survival in CRC [17]. Mechanistically, MRTFB is activated primarily through Rho-dependent actin polymerization. In the resting state, MRTFB is sequestered in the cytoplasm and associates with unpolymerized G-actin through its RPEL motifs. When extracellular signals stimulate the Rho pathway, Rho GTPases promote the polymerization of actin into filamentous F-actin via downstream effectors such as ROCK, LIMK, and mDia, thereby decreasing G-actin. This decrease leads to the dissociation of MRTFB from G-actin, allowing it to translocate into the nucleus. Inside the nucleus, MRTFB acts as a transcriptional coactivator for SRF and is involved in Rho-actin signaling pathways [18,19].

*MRTFB* is associated with several diseases, including cancer, neurodevelopmental disorders, and cardiovascular disorders. In the context of cancer, MRTFB exhibits context-dependent activity: it acts as a tumor suppressor in gastrointestinal (GI) tract cancers such as colorectal cancer (CRC), hepatocellular carcinoma (HCC), and pancreatic ductal adenocarcinoma (PDAC) [17,20,21]. In CRC, lower expression of MRTFB by RNA interference results in increased cell invasion and migration, leading to shorter patient survival [17]. Conversely, MRTFB also acts as an oncogene in breast cancer by promoting cancer metastasis , and in pancreatic cancer, overexpression of MRTFA/B promotes epithelial–mesenchymal transition (EMT) and gemcitabine resistance [22]. Other cancers associated with MRTFB include chondroid lipoma and ectomesenchymal chondromyxoid tumor. The chondroid lipoma is a rare soft tissue tumor characterized by recurrent *ZFTA* (C11orf95)::*MRTFB* fusion [23], whereas the ectomesenchymal chondromyxoid tumor is a fusion of *RREB1*::*MRTFB* gene [24]. The de novo variants p.R104G and p.A91P in MRTFB have been associated with neurodevelopmental disorders. They act as gain-of-function mutations that reduce actin binding, thereby increasing gene transcription [12]. Moreover, MRTFB is also involved in cardiovascular disorders such as vascular malformations, heart failure, and thoracic aortic dissection [14,25,26]. The global knockout of the *MRTFB* gene in mice causes vascular malformations, particularly deformation of the aortic arch arteries and defective smooth muscle cell differentiation, resulting in embryonic lethality [26]. Deletion of cardiac-specific MRTFA/B alleles in mice results in decreased cardiac contractility and adult-onset heart failure due to sarcomere disarray [25]. Mice lacking MRTFB in hair cells develop early-onset hearing loss, disrupted hair bundle morphology, and structural abnormalities of the actin cytoskeleton independently of SRF. The target genes affected by MRTFB deficiency are Nox4 and Kif14, which are not regulated by SRF, suggesting an SRF-independent pathway of action for MRTFB in hair cells [27].

A few small-molecule inhibitors have been developed to pharmacologically target MRTF-associated pathways. The first-generation Rho/MRTF/SRF pathway inhibitor CCG-1423 was found to prevent the nuclear translocation of MRTFA/B by inhibiting their interaction with importin α/β1. CCG-1423-mediated depletion of nuclear MRTF repressed α-SMA protein expression and TGF-β-induced fibrogenesis; however, it was highly cytotoxic [28]. The second-generation inhibitors include CCG-100602, CCG-203971, and CCG-232601 [28,29]. In human colonic myofibroblasts, both CCG-100602 and CCG-203971 inhibited matrix stiffness- and TGF-β-induced fibrogenesis, while exhibiting lower cytotoxicity than the first-generation inhibitor [28]. Patyal P et al. (2024) demonstrated that CCG-203971 and CCG-232601 inhibit RhoA, MRTF-A, and MRTF-B downstream signaling involving transcription factor SRF and co-factor p49/STRAP in normal cell lines of human lung fibroblasts (WI-38) and mouse myoblasts (C2C12) [29].

Despite its established role in actin cytoskeletal dynamics, the site-specific phosphoregulatory landscape of MRTFB, including its upstream kinases and co-regulated protein interactors, remains poorly identified. Here, we performed a systematic integration of publicly available phosphoproteomics studies to identify the predominant phosphosites of MRTFB and to predict its upstream kinases, interacting partners, and protein complexes using a co-regulation-based strategy. In addition, we assessed the conservation of MRTFB-predominant phosphosites across species and among the MRTF family members. Furthermore, co-occurrence patterns of predominant phosphosites, as well as the signaling pathways associated with co-regulated proteins, were explored.

## 2. Materials and Methods

### 2.1. A Comprehensive Screening and Assembly of MRTFB Phosphosites from Global Phosphoproteomics Datasets

To curate the phosphoproteomics datasets, we conducted a PubMed query search using the terms “phosphoproteomics” OR “phosphoproteome” while excluding “Plant” and “Review”. We manually screened the academic literature to assemble high-confidence phosphoproteomics datasets from human cellular datasets containing class-1 MRTFB phosphosites, requiring a localization probability > 75% and an A-score > 13. These datasets were classified according to their enrichment methods (STY or ST, or Y phosphosites) and categorized into quantitative differential datasets (comparing test vs. control conditions) and qualitative profiling datasets (experimental and control conditions are recorded as independent phosphosite inventories). For the differential analysis of individual datasets, we applied a *p*-value cutoff of <0.05 and a fold-change threshold of >1.3 for upregulation and <0.76 for downregulation. To maintain uniform mapping, each phosphosite was mapped according to its gene symbol as per HGNC (HUGO Gene Nomenclature Committee) (downloaded on 30 May 2023) and UniProt (13 April 2023) accessions using our in-built mapping tool.

### 2.2. Mapping the Predominant MRTFB Phosphosites from Global Phosphoproteomics Datasets

We utilized class-1 phosphosites for all downstream analyses. For each site, we calculated detection frequencies and ranked identification frequencies across both qualitative profiles and quantitative differential datasets. Phosphosites exhibiting the highest frequency across all datasets were defined as predominant phosphosites. Specifically, the highest-frequency phosphosites were detected in over 18% of the combined qualitative and quantitative datasets. Notably, as this study focuses exclusively on high-confidence, mass-spectrometry-derived class-1 phosphosites, sites traditionally identified by phospho-specific antibodies or site-directed mutagenesis were not included.

### 2.3. Filtering for Phosphosites Synchronously Regulated with Predominant MRTFB Phosphosites

To identify phosphosites in other proteins (PsOPs) that exhibit positive or negative co-regulation with the predominant MRTFB sites (S66 and S921), we analyzed their corresponding quantitative differential expression datasets. Given the heterogeneity of the experimental conditions and platforms used across the source studies, raw data re-analysis was not feasible; hence, we utilized the data reported in the original articles. To identify synchronously regulated phosphosites, we examined instances in which the MRTFB phosphosite was upregulated while the associated PsOPs were either upregulated or downregulated, which were classified as UU and UD, respectively. Conversely, when the MRTFB phosphosite was downregulated, the associated upregulated or downregulated PsOPs were classified as DU and DD, respectively. Later, they were paired as UUDD (positive co-regulation) and UDDU (negative co-regulation).

We further analyzed the data using a one-sided Fisher’s exact test (FET), following an established contingency table [30,31,32,33]. The FET *p*-value was calculated using the formula:

$$\sum p=\frac{\left(a+b\right)!\left(c+d\right)!\left(a+c\right)!\left(b+d\right)!}{n!}\;\sum_{i}\frac{1}{a_{i}!b_{i}!c_{i}!d_{i}!}$$

Here, “*a*” represents neither site detected, “*b*” represents only one site detected, “*c*” represents both sites detected with opposite regulation patterns, and “*d*” represents both sites detected with the same regulation pattern. To identify high-confidence phosphosite pairs, we implemented a rigorous filtration process based on four criteria. First, we set a significant threshold of *p*-value < 0.05. Second, we established a 10% ratio threshold for both positive (n_UUDD/n_UDDU) and negative (n_UDDU/n_UUDD) co-regulation. To ensure biological and experimental reproducibility, we mandated that each pair be identified in at least three separate publications (PMID_confidence) and across three different experimental conditions (Code_confidence). These high-confidence phosphosite pairs were used for further analysis.

### 2.4. Analysis of Functionally Interdependent MRTFB Phosphosites

Functionally associated phosphorylation sites often co-occur at a frequency significantly higher than random chance, suggesting that they may be sequentially or structurally adjacent, phosphorylated by the same kinases, or involved in shared regulatory pathways [34]. To explore such mutually associated phosphosites within MRTFB, we scrutinized the patterns of co-differential regulation across all identified phosphosite pairs. We specifically separated the differential datasets in which multiple MRTFB sites were recorded under identical experimental conditions. For each pair, the frequencies of UUDD and UDDU co-regulation were calculated separately. The degree of functional association was then quantified by calculating the ratios of positive co-regulation (n_UUDD/n_UDDU) and negative co-regulation (n_UDDU/n_UUDD).

### 2.5. Analysis of the MRTFB Interaction Network

To analyze the MRTFB interactomes, we compiled a comprehensive list of binary and complex protein–protein interactors from several curated repositories. It includes primary interaction databases such as HPRD (Human Protein Reference Database, Institute of Bioinformatics, Bangalore, India) [35], BioGRID (Biological General Repository for Interaction Datasets, Toronto, Ontario, Canada) [36], BIND (Biomolecular Interaction Network Database, Blueprint Initiative, Toronto, Ontario, Canada) [37], and IntAct (IntAct Molecular Interaction Database, Cambridge, United Kingdom) [38], as well as integrated databases including ConsensusPathDb (Release 35, Berlin, Germany) (downloaded on 22 May 2023) [39]. Other databases include CORUM (comprehensive resource of mammalian protein complexes, Neuherberg) (downloaded on 3 March 2023) [40], specifically for mammalian protein complexes, and RegPhos 2.0 (Regulatory Network in Protein Phosphorylation, Taiwan, China) (downloaded on 24 May 2023) [41], for phosphorylation-dependent regulatory networks.

### 2.6. Integration of Predictive and Experimental Kinase Networks

To identify the potential upstream kinases of MRTFB, we integrated predicted upstream kinase data from various sources. The in silico predicted kinases were obtained from NetworKIN (a resource for exploring cellular phosphorylation networks, Samuel Lunenfeld Research Institute, Mount Sinai Hospital, Toronto, Canada) (downloaded on 4 January 2023) [42] and AKID (Automatic Kinase-specific Interactions Detection, Centro di Bioinformatica Molecolare, University of Rome “Tor Vergata”, Rome, Italy) (downloaded on 24 May 2023) [43], which utilize sequence motifs and cellular context to identify potential phosphorylation events. These predictions were cross-referenced with experimentally validated kinase-to-phosphosite relationships retrieved from iKiP-DB (in vitro kinase-to-phosphosite database, Berlin) [44]. Furthermore, we incorporated a high-confidence kinase-substrate dataset identified via synthetic peptide library screening by Johnson et al. (2023) [45], applying a stringent percentile cutoff of ≥90%.

### 2.7. Intrinsically Disordered Region Prediction

The intrinsically disordered regions (IDRs) of MRTFB were predicted using IUPred3 (https://iupred3.elte.hu/) (Eötvös Loránd University, Pázmány Péter stny 1/c, Budapest H-1117, Hungary) [46]. The protein sequences in FASTA format were obtained from UniProt (https://www.uniprot.org/) (EMBL-EBI, Wellcome Genome Campus, Hinxton CB10 1SD, United Kingdom) [10]. IUPred3 uses default parameters, including “IUPred3 long disorder” in analysis type, anchoring strength 0.5, and a 21-residue window size for smoothing. The output scores were ranged from 0 for ordered to 1 for disordered regions, at a threshold of 0.5 to classify disordered (>0.5) and ordered (<0.5) regions [47].

### 2.8. Gene Enrichment Analysis

The pathway enrichment analysis was performed using ShinyGO (https://bioinformatics.sdstate.edu/go/) (South Dakota State University) [48]. KEGG pathway database (https://www.genome.jp/kegg/pathway.html) (Kyoto, Japan) (accessed on 8 June 2026) [49] was used to obtain the pathway map. The functional enrichment analysis was conducted using the STRING database (https://string-db.org/) [50].

### 2.9. Conservation Analysis

Multiple sequence alignment was performed using Clustal Omega (https://www.ebi.ac.uk/jdispatcher/msa/clustalo) (EMBL-EBI, Wellcome Genome Campus, Hinxton CB10 1SD, United Kingdom) (accessed on 26 June 2026) [51] to assess the conservation of MRTFB phosphosites across species and among MRTF family members. The protein FASTA sequences were obtained from UniProt (https://www.uniprot.org/) [10].

### 2.10. Data Visualization

The lollipop plot was generated using the M2Viz tool (https://www.ciods.in/m2viz) (CIODS, Yenepoya (Deemed to be University, Mangalore, Karnataka, India)) (accessed on 8 June 2026) [52]. Cytoscape [53] was used for the interaction map visualization, and RAWGraph 2.0 (Politecnico di Milano (DensityDesign Research Lab), Italy) (https://doi.org/10.1145/3125571.3125585) was used to create the dendrogram and bar graph.

## 3. Results

### 3.1. Assembly of Class-1 Phosphosites of MRTFB

We screened over 3825 published studies on global human phosphoproteomics and identified 541 profiling and 167 differential datasets containing class-1 phosphosites to analyze functionally relevant phosphosites in MRTFB (Table S1A,B). The phosphorylation was identified at 58 residues in the profiling datasets and at 26 residues in the differential datasets. Of the 58 phosphosites identified in the profiling dataset, 10 have not been reported in PhosphoSitePlus [54]. These include S92, S97, S200, T215, T217, T227, T399, S495, T536, and T545. Such insights from mass spectrometry-based phosphoproteome analysis of profiling datasets help us discover previously unknown phosphorylation sites that serve regulatory roles in cellular function. In contrast, MRTFB phosphosites such as S76, T82, T170, S175, S185, S214, T257, S376, S490, N537, S632, S823, N840, and S1041 were not identified in our phosphoproteomic datasets of class-1 phosphosites. It indicates the importance of combining both high- and low-throughput data to detect and validate the function of protein phosphorylation sites.

### 3.2. Identification of Predominant Phosphosites of MRTFB

**Figure 1:**
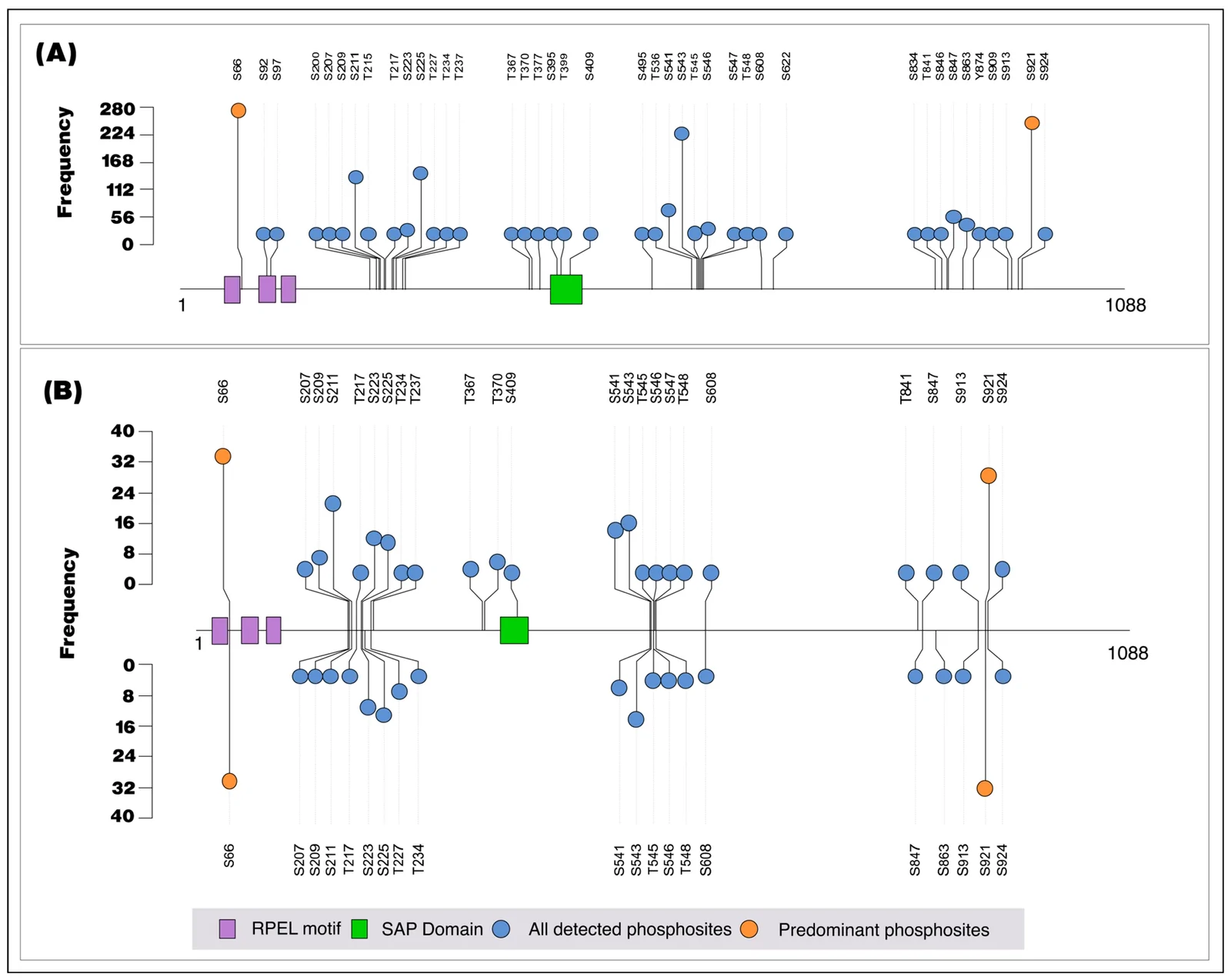
Lollipop plot demonstrating the phosphosites and predominant sites of MRTFB identified from mass spectrometry-based global human cellular phosphoproteomic profiling and differential expression datasets. The X-axis denotes the amino acid sequence length, and the Y-axis denotes the frequency of phosphosites. The predominant phosphosites are highlighted in orange. (**A**) Class-1 phosphosites of MRTFB in the profiling dataset. (**B**) Class-1 phosphosites of MRTFB in the differential expression dataset.

To determine biologically relevant phosphosites, we prioritized phosphosites based on their detection frequency across diverse experimental and biological conditions. Both profiling and differential phosphoproteomic datasets were analyzed, and phosphosites were ranked according to their frequency of detection. Frequently detected phosphosites are more likely to be associated with functionally significant sites. MRTFB phosphosites, such as S66 and S921, were the most frequently detected, with frequencies of 65 and 62, respectively. Thus, we considered them as predominant phosphosites of MRTFB. The class-1 phosphosites and predominant sites of MRTFB identified from the global human cellular profiling and differential datasets are represented in Figure 1. Consistent with this, both sites have been recurrently reported in PhosphoSitePlus, supporting their reproducible detection across studies.

**Figure 2:**
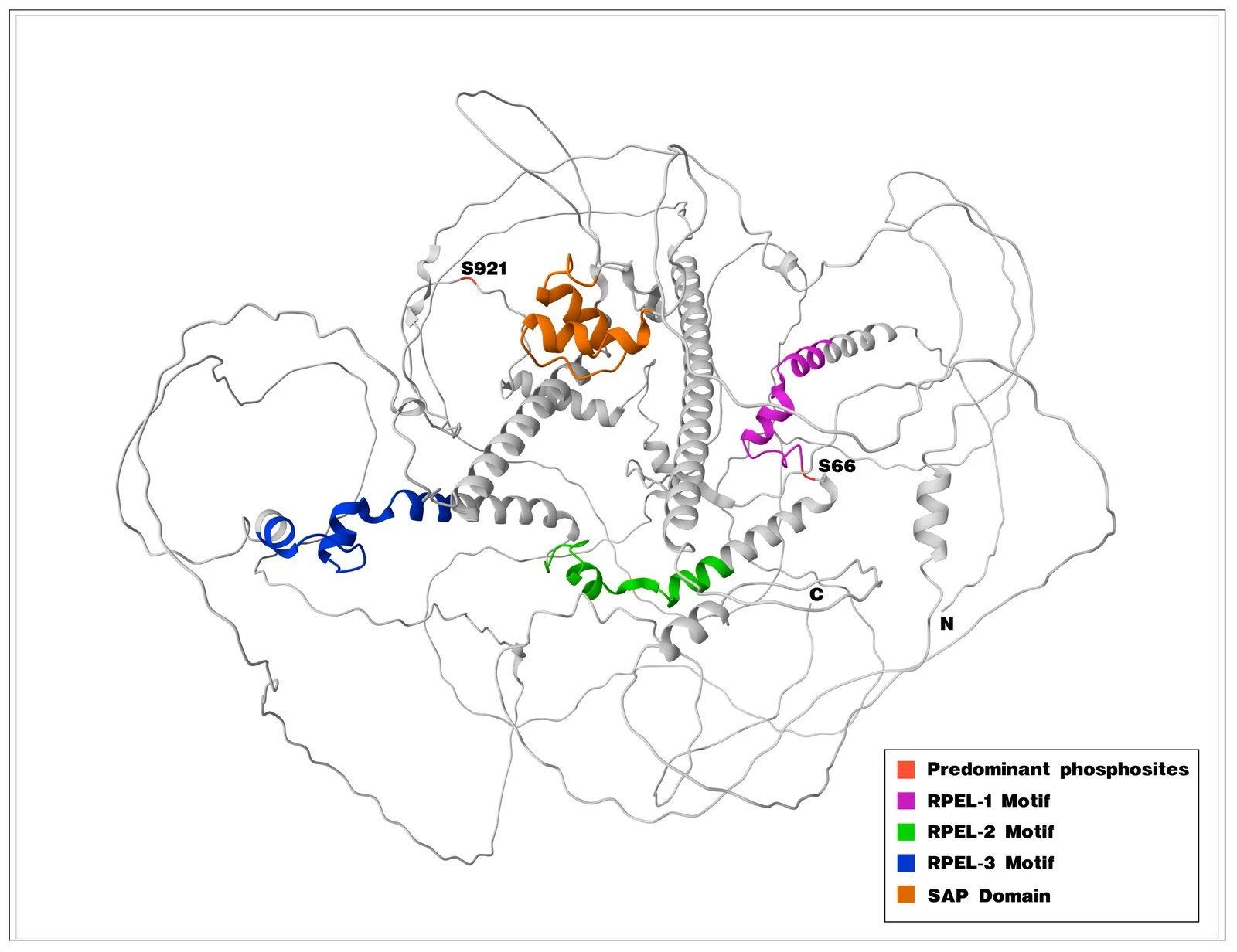
Alpha fold structure of MRTFB protein with its motifs, domain, and predominant phosphosites highlighted. The predominant phosphosites, S66 and S921, are highlighted in red, the RPEL-1 motif next to the predominant site S66 is highlighted in purple, the RPEL-2 motif is highlighted in green, the RPEL-3 motif is highlighted in blue, and the SAP domain is highlighted in orange.

**Figure 3:**
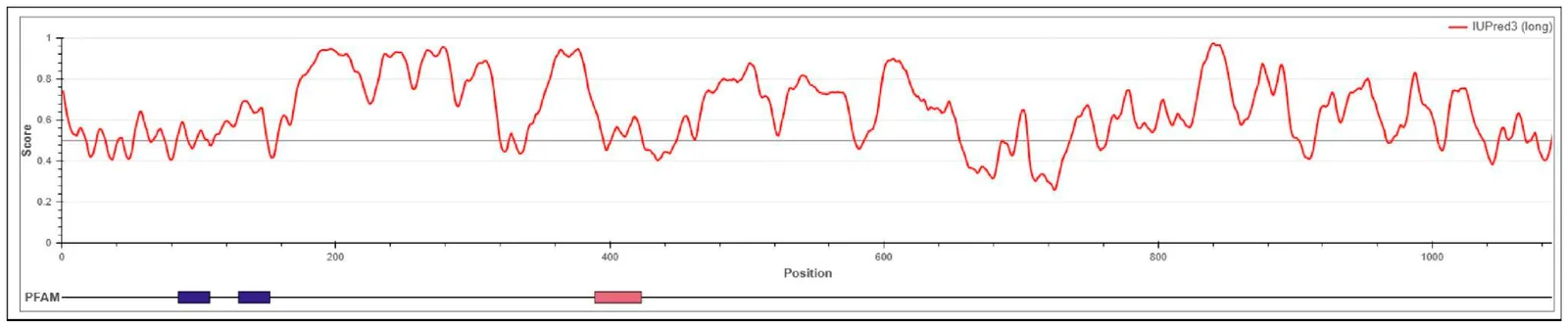
Intrinsically disordered regions of human MRTFB protein. The X-axis shows the amino acid position, and the Y-axis shows the IUPred3 (long disorder) prediction score (red line). The scores >0.5 denote the regions that are likely to be intrinsically disordered, and the scores <0.5 indicate ordered regions. The blue box represents RPEL motifs, the red box represents the SAP domain, and the red line represents IUPred3 long disorders.

Structurally, the phosphorylation site S66 of MRTFB is situated adjacent to the RPEL-1 motif (40–65) and is important for nucleo-cytoplasmic shuttling [6,55], while the phosphosite S921 is present at the C-terminal region (701–1049), which has a transcriptional activation domain that is regulated by its N-terminal domain [6] (Figure 2). The experimental validation using a phospho-null mutant (S66A) demonstrates that the specific phosphorylation event at S66 is essential for MRTF-B to dissociate from G-actin and accumulate in the nucleus. Consequently, this Serine 66-mediated translocation drives rapid activation of SRF-dependent gene transcription, such as the expression of *Fos*, in response to mechanical muscle activity [55]. The intrinsically disordered region (IDR) prediction of MRTFB by IUPred3 indicates that the sequence from 914 to 967 (53 residues) lies in regions with scores above the disorder threshold (~0.5) (Figure 3). The predominant phosphosite, S921, lies within this sequence, suggesting that these residues are located in weakly disordered or flexible regions, which may facilitate regulatory phosphorylation [46,56].

### 3.3. Co-Regulation Analysis of Predominant Sites of MRTFB

**Figure 4:**
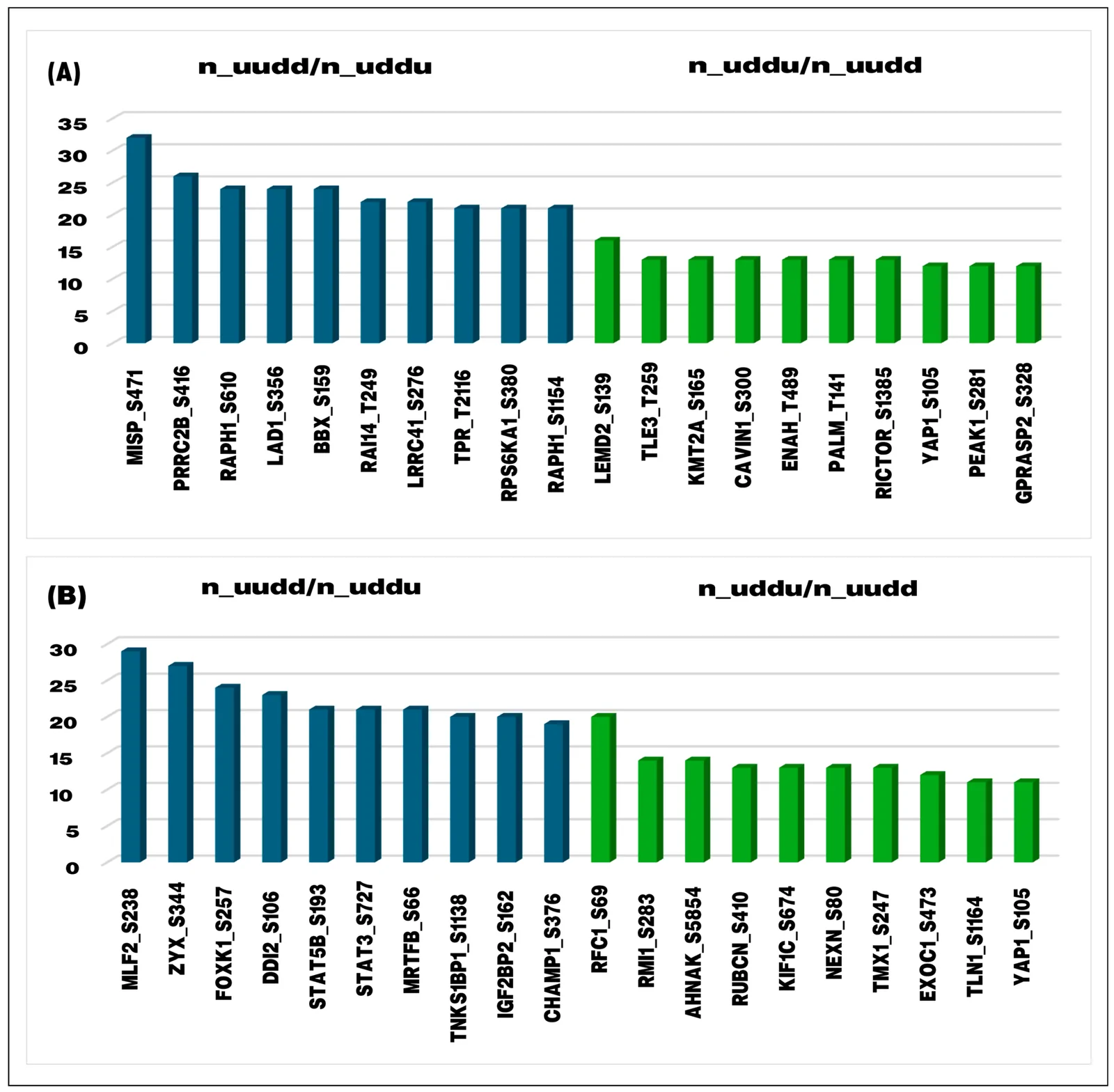
Bar diagram illustrating the top 10 PsOPs that showed positive and negative co-regulation with the predominant sites of MRTFB. The X-axis denotes the co-regulated PsOPs, and the Y-axis denotes the frequency of phosphosites. The blue bars denote the positively co-regulated PsOPs, and the green bars denote the negatively co-regulated PsOPs. (**A**) Top 10 positively and negatively co-regulated PsOPs of MRTFB at S66. (**B**) Top 10 positively and negatively co-regulated PsOPs of MRTFB at S921.

We performed a co-regulation analysis to determine the phospho-signaling pattern of PsOPs associated with the predominant phosphosites of MRTFB. We identified PsOPs that consistently exhibit expression co-regulation with predominant sites. To ensure the biological and statistical significance of these data, we applied strict criteria based on the FET contingency table. The criteria include a *p*-value of less than 0.05, a 10% frequency cutoff for differential frequency of predominant sites for both positive and negative co-regulation, validation across at least three independent publications (PMID confidence), and three distinct experimental conditions (code confidence). After applying these criteria, the predominant site S66 of MRTFB showed 538 and 144 PsOPs that are positively and negatively co-regulated, respectively (Table S2A,B). Likewise, the site S921 showed 658 and 116 PsOPs with positive and negative co-regulation (Table S2C,D). The top 10 PsOPs that are positively and negatively co-regulated with the predominant sites of MRTFB are represented in Figure 4.

**Figure 5:**
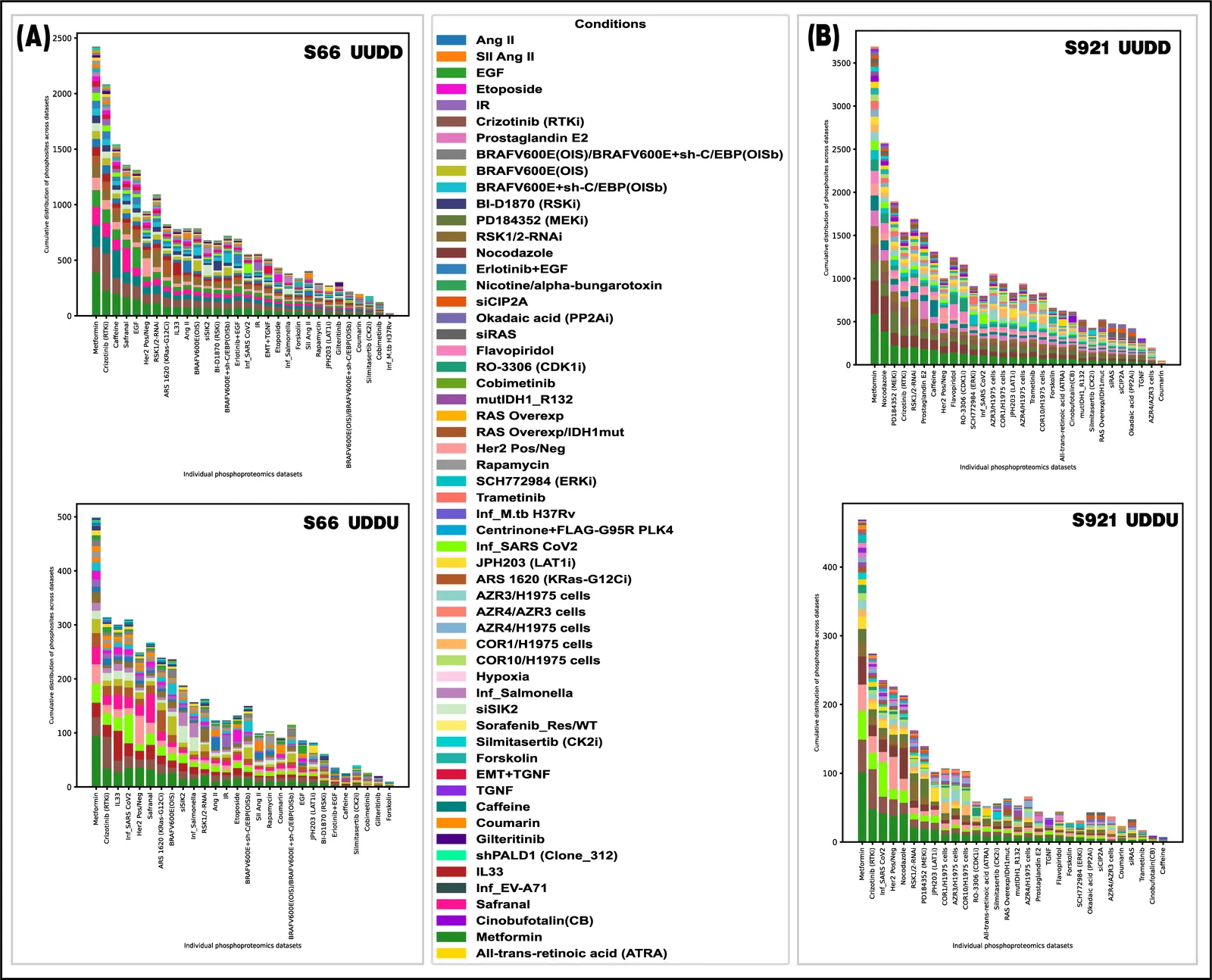
Cumulative distribution plot of the expression of predominant phosphosites of MRTFB across different experimental conditions. (**A**) Experimental conditions of positively and negatively co-regulated PsOPs of phosphosite S66. (**B**) Experimental conditions of positively and negatively co-regulated PsOPs of phosphosite S921.

Among the 538 positive PsOPs of S66, MISP_S471 showed top co-regulation with a frequency of 32, and from 144 negative PsOPs, LEMD2_S139 showed top co-regulation with a frequency of 16. MISP (mitotic interactor and substrate of Plk1), also known as Caprice and C19orf21, is an actin-bundling protein that has an important role in actin-based cytoskeletal reorganization. The phosphorylation of S471 in MISP decreases its affinity for actin, indicating involvement in mitotic reorganization of the actin cytoskeleton [57]. The LEMD2 (LEM-domain containing 2) plays an important role in maintaining the nuclear envelope (NE) integrity and interacts with the LINC (Linker of Nucleoskeleton and Cytoskeleton) complex [58]. Similarly, for the site S921, among its 658 positive PsOPs, MLF2_S238 showed top co-regulation with a frequency of 29, and from 116 negative PsOPs, RFC1_S69 showed top co-regulation with a frequency of 20. MLF2 (myeloid leukemia factor 2) acts as an oncogene in breast cancer and promotes tumor initiation and metastasis [59,60]. RFC1 (replication factor C subunit 1) is associated with neurodegenerative disorders such as cerebellar ataxia, neuropathy, and vestibular areflexia syndrome (CANVAS) [61,62]. However, further phosphorylation-related studies are required to assess the relation between these PsOPs. The expression of predominant phosphosites of MRTFB across different experimental conditions is illustrated in Figure 5.

### 3.4. Kinases and Phosphatases Co-Regulated with Predominant MRTFB Phosphosites

**Figure 6:**
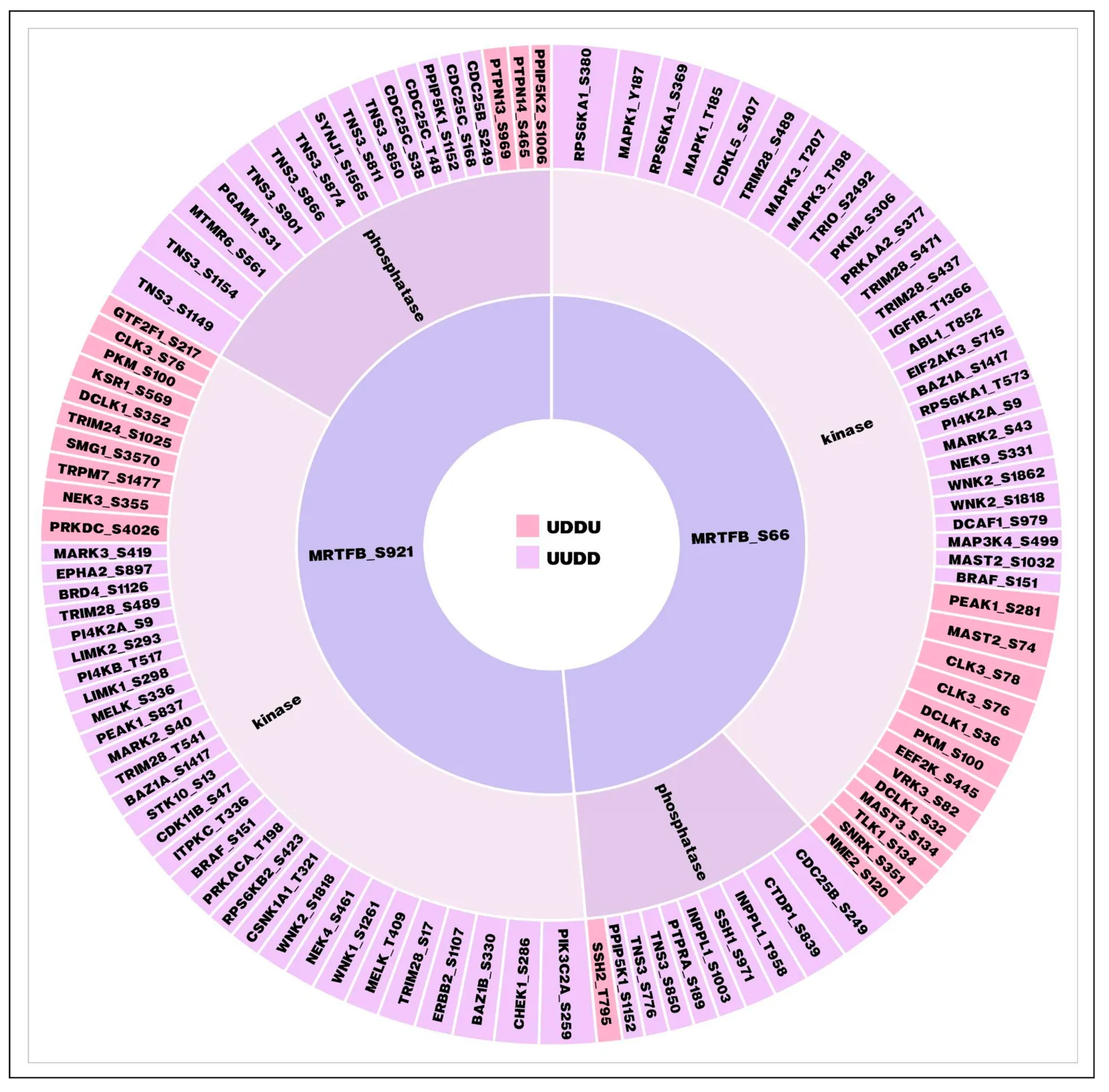
Interaction network of predominant sites of MRTFB interacting with kinases and phosphatases. The PsOPs that are positively co-regulated (violet) and negatively co-regulated (pink) with MRTFB-predominant phosphosites in the kinases and phosphatases.

The current research often focuses on upstream kinases, substrates, or interactors, while the complex crosstalk among multiple signaling modules across various biological conditions is rarely visualized on a global scale. In our study, we assembled global phosphoproteomic datasets to provide a specific platform for identifying and visualizing these co-regulated proteins at the phosphosite level. By categorizing the phosphosites in kinases and phosphatases that are positively and negatively co-regulated with the predominant sites of MRTFB, we were able to establish an interacting signaling network. The overall network of MRTFB predominant sites interacting with phosphosites in kinases and phosphatases is illustrated in Figure 6.

The predominant MRTFB phosphosite, S66, was positively co-regulated with the phosphosites in RPS6KA1 (S380, T573) and MAPK1 (Y187, T185), which are associated with inducing kinase activity [63,64], while the PsOP, MAPK3_T207, has been associated with inhibition of kinase activity [65]. Additionally, MRTFB was positively co-regulated with phosphosites in several kinases, including RPS6KA1_S369, CDKL5_S407, TRIM28_S489, S437 and S471, MAPK3_T198, TRIO_S2492, PKN2_S306, PRKAA2_S377, IGF1R_T1366, ABL1_T852, EIF2AK3_S715, BAZ1A_S1417, PI4K2A_S9, MARK2_S43, NEK9_S331, WNK2_S1862 and S1818, DCAF1_S979, MAP3K4_S499, MAST2_S1032, and BRAF_S151, which are not associated with kinase activity. The phosphosites in phosphatases, such as CDC25B_S249, CTDP1_S839, INPPL1_T958, SSH1_S971, INPPL1_S1003, PTPRA_S189, TNS3_S850 and S776, and PPIP5K1_S1152, are also currently not associated with enzymatic activity. The site S66 of MRTFB was negatively co-regulated with kinases, such as PEAK1_S281, MAST2_S74, CLK3_S78 and S76, DCLK1_S36 and S32, PKM_S100, EEF2K_S445, VRK3_S82, MAST3_S134, TLK1_S134, SNRK_S351, and NME2_S120, as well as the phosphatase SSH2_T795, whose functional roles in regulating enzymatic activity have not yet been characterized.

Likewise, the S921 site of MRTFB exhibited positive co-regulation with kinase enzymatic activity-inducing phosphosites in WNK1 (S1261) and RPS6KB2(S423), as well as enzymatic activity-inhibiting phosphosites in the phosphatase CDC25C (S168) [66,67,68]. MRTFB S921 also showed positive co-regulation with kinases such as PIK3C2A_S259, CHEK1_S286, BAZ1B_S330, ERBB2_S1107, TRIM28_S17, S489 and T541, MELK_T409 and S336, NEK4_S461, WNK2_S1818, CSNK1A1_T321, BRAF_S151, ITPKC_T336, CDK11B_S47, STK10_S13, BAZ1A_S1417, MARK2_S40, PEAK1_S837, LIMK1_S298, PI4KB_T517, LIMK2_S293, PI4K2A_S9, BRD4_S1126, EPHA2_S897, and MARK3_S419 and phosphatases such as TNS3_S1149, S1154, S901, S866, S874, S811 and S850, MTMR6_S561, PGAM1_S31, SYNJ1_S1565, CDC25C_S38 and T48, PPIP5K1_S1152, and CDC25B_S249. The S921 was negatively co-regulated with phosphosites in kinases such as PRKDC_S4026, NEK3_S355, TRPM7_S1477, SMG1_S3570, TRIM24_S1025, DCLK1_S352, KSR1_S569, PKM_S100, CLK3_S76, and GTF2F1_S217 and phosphatases such as PPIP5K2_S1006, PTPN13_S969, and PTPN14_S465; these sites are currently not associated with enzymatic activity.

**Figure 7:**
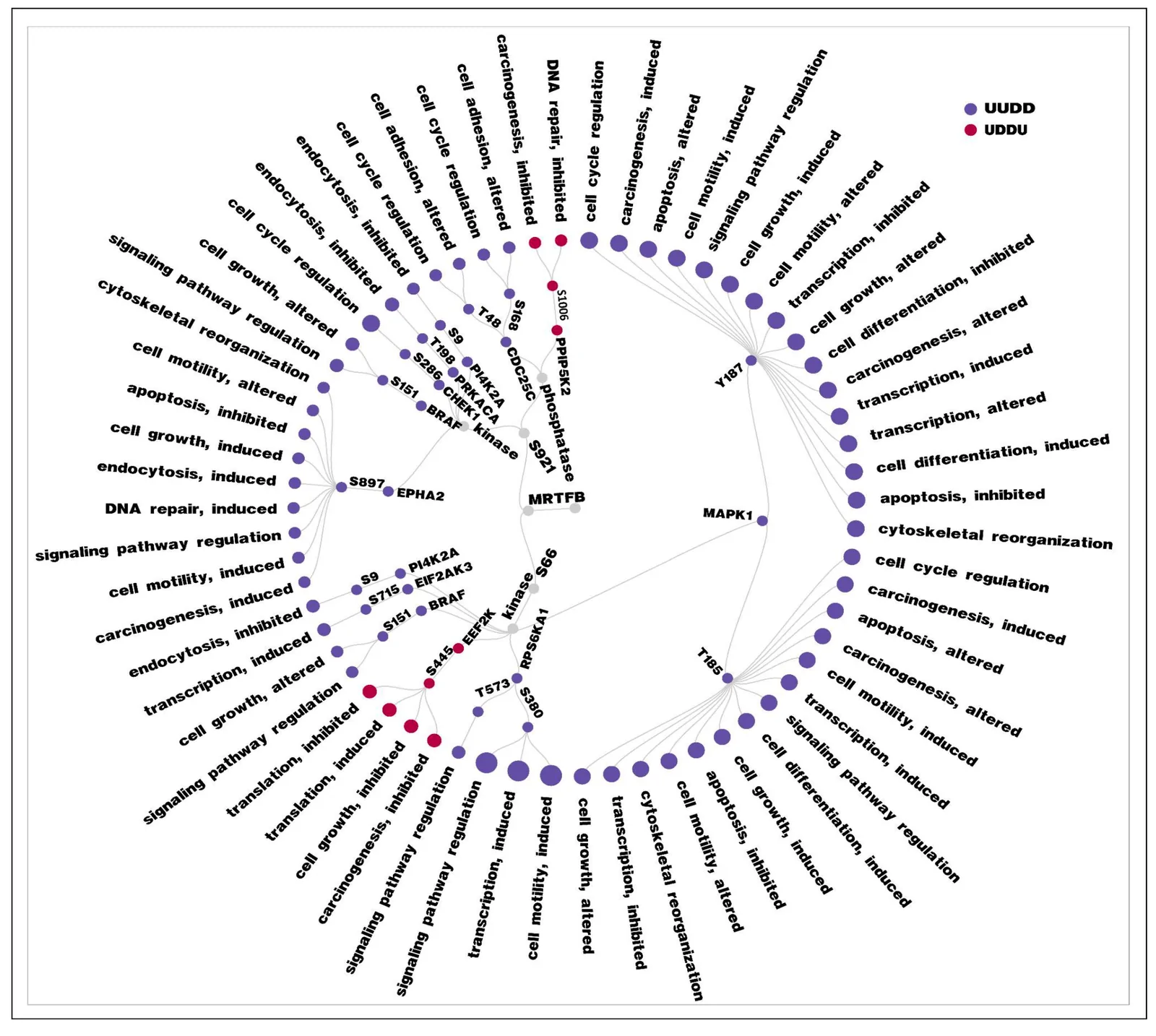
Interaction network of predominant sites of MRTFB interacting with kinases and phosphatases with phosphosite-level annotation of their regulatory association with biological processes and enzymatic activity. The PsOPs and biological processes that are positively co-regulated (purple) and negatively co-regulated (red) with MRTFB and predominant sites of MRTFB in the kinases and phosphatases.

Although these phosphosites are discussed here primarily in the context of their enzymatic activity, several distinct biological processes are also associated with them as functional signature phosphosites (Figure 7). In our study, we identified signature phosphosites in 66 kinases and 18 phosphatases that are positively or negatively co-regulated with the predominant MRTFB phosphosites. The kinase–phosphatase phosphosite level co-expression network in this study provides a platform for analyzing phosphoproteomic datasets containing differential regulation of MRTFB phosphosites.

### 3.5. Co-Occurrence Patterns of Phosphosites Within MRTFB

**Figure 8:**
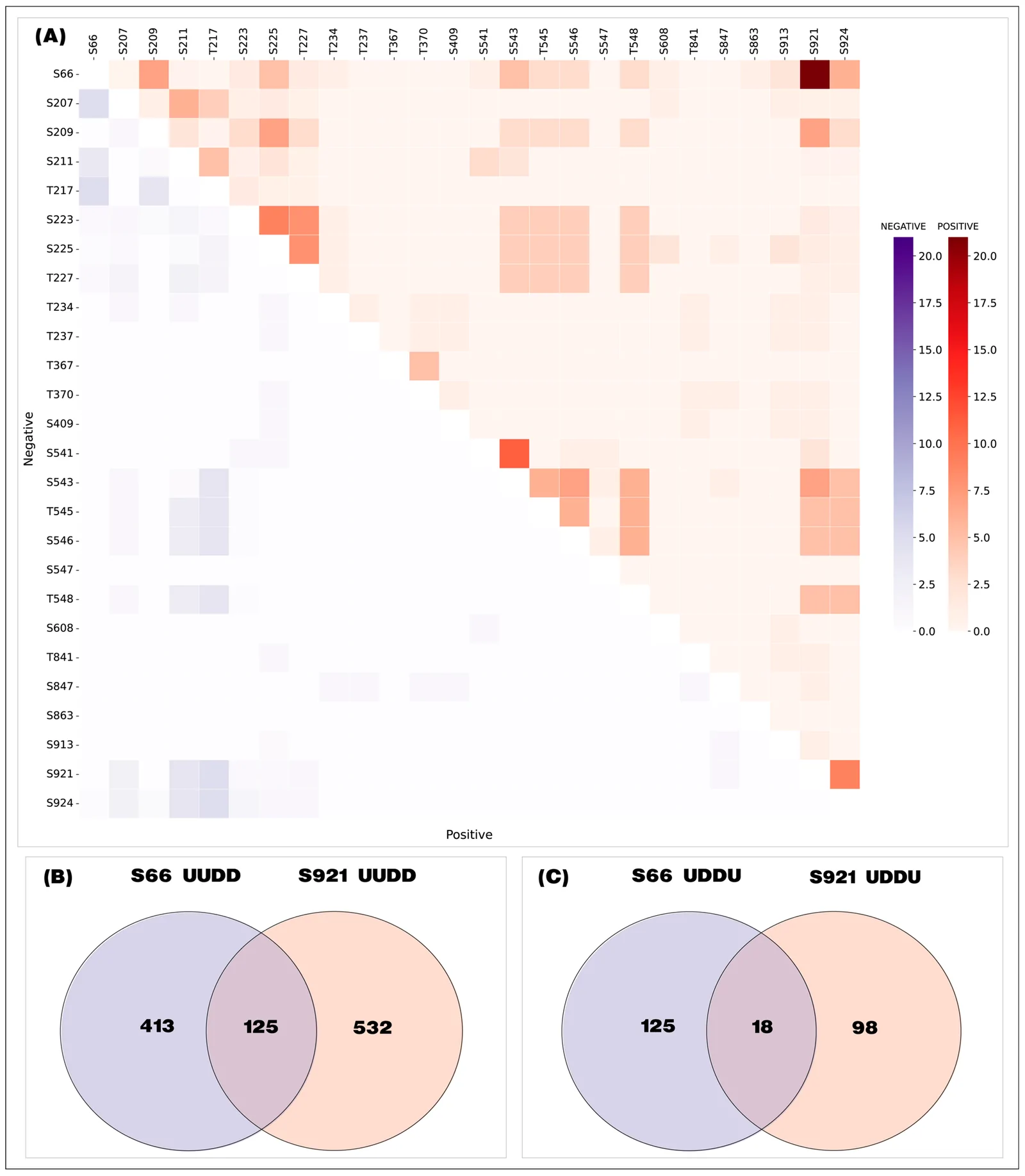
Co-occurring phosphosite pairs within MRTFB. (**A**) Heatmap representing the co-occurrence pattern of phosphosites within MRTFB. The upper red triangle denotes the positive co-occurrence pattern, and the lower violet triangle denotes the negative co-occurrence pattern between phosphosite pairs of MRTFB, with color gradient representing the frequency of co-occurrence. (**B**) Venn diagram illustrating the number of PsOPs that are commonly positively co-regulated with the predominant sites S66 and S921 of MRTFB. (**C**) Venn diagram illustrating the number of PsOPs that are commonly negatively co-regulated with the predominant sites S66 and S921 of MRTFB.

A qualitative co-occurrence analysis method introduced by Li et al. (2017) [34] demonstrated that phosphorylation site pairs co-expressed across various mass spectrometry datasets show a higher probability of functional association than random pairs. Their methodology demonstrates that these co-occurring sites often participate in the same signaling pathways or regulatory modules. To further explore these relations, we assessed the co-occurrence profiles of individual phosphosites within our integrated global datasets, specifically focusing on the differential phosphoproteomic datasets of MRTFB (Figure 8A). Additionally, we categorized the datasets into two groups: those that showed positive co-occurrence, where phosphosites were both co-regulated or downregulated, and those showing negative co-occurrence, where one site was upregulated while the other was downregulated. From the data, we identified that the predominant sites S66 and S921 of MRTFB show top positive co-occurrence. They were upregulated together in 21 datasets and downregulated together in 1 dataset, with a ratio of 21 (Table S3A).

### 3.6. Comparative Analysis of S66 and S921 Phosphosites

A comparative analysis method was used to analyze functional overlap of PsOPs of predominant phosphosites S66 and S921. Among them, we identified 125 PsOPs that are commonly positively co-regulated (Figure 8B). Notably, SOS1 and BRAF are central mediators of the MAPK signaling pathway, while STAT3 acts as a mediator of resistance to MAPK pathway inhibitors [69,70,71,72]. The phosphorylation of SOS1 at 1210 is an Ras pathway component targeted by JNK in rigosertib-treated cells, while phosphorylation of STAT3 at S727 enhances its transcriptional activity and mitochondrial function [73,74]. MRTFA serves as a transcriptional co-activator that works alongside MRTFB to drive SRF-mediated gene expressions in response to Rho-GTPase signaling and actin dynamics [19]. ARHGEF5 (also known as TIM) and ARHGEF12 (also known as LARG/ARHGEF12) are GEFs that activate RhoA [75,76]. The active RhoA is the primary activator of the MRTF family (including MRTFB). When RhoA is activated, actin polymerization occurs, reducing G-actin levels and freeing MRTF to enter the nucleus to activate target genes [77]]. PPP1R9B (spinophilin) and PPP1R12C (MYPT3) are subunits of protein phosphatase 1 (PP1) that modulate actin–myosin contractility [78]. The PPP1R9B at S94 is a site for PKA phosphorylation that regulates its interaction with F-actin [79].

Among the co-regulated PsOPs of predominant sites S66 and S921, we identified 18 PsOPs that are commonly negatively co-regulated (Figure 8C). Among these, YAP1, TLN1, and RICTOR are critical regulators of mechanotransduction and regulate cell growth, proliferation, and differentiation [80,81,82,83]. YAP1 acts as a primary transcriptional coactivator in the Hippo pathway, translocating to the nucleus to drive genes involved in proliferation and survival [84]. The TLN1 (Talin-1) acts as a mechanical link between integrins and the actin cytoskeleton, essential for focal adhesion assembly [85]. RICTOR is a crucial component of the mTORC2 complex, essential for regulating AKT phosphorylation and involved in cell migration, proliferation, survival, and cytoskeletal rearrangement [80]. DLC1 acts as a RhoGAP that regulates RhoA activity, influencing cytoskeletal organization and cell morphology [86].

Since the predominant phosphosites S66 and S921 of MRTFB show strong positive co-occurrence and many PsOPs that are commonly co-regulated, it suggests that they might have potential co-regulation within shared or converging signaling networks.

### 3.7. Systematic Cataloging of Potential Upstream Regulatory Kinases Functionally Associated with MRTFB

Currently, no experimentally validated upstream kinases are known to phosphorylate the predominant phosphosites of MRTFB. To identify upstream kinases that phosphorylate predominant sites of MRTFB, we integrated the kinase–substrate data from Johnson et al. (2023) [45] and data obtained from predictive tools such as NetworKIN [42] and AKID [43]. After screening the data, we identified phosphosites in two predicted upstream kinases (MAPK1_T185/Y187 and MAPK3_T207/T198) that positively co-regulated with the predominant site S66 of MRTFB. Similarly, S66 of MRTFB shows one predicted upstream kinase (MAST3_S134) that is negatively co-regulated. For S921, it also shows one predicted upstream kinase (CSNK1A1_T321) that is positively co-regulated with MRTFB (Table S3B).

MAPK1 and MAPK3 function as key upstream regulatory kinases of MRTFB predicted by Johnson et al. (2023) [45]. Both MAPK1 and MAPK3 are involved in nuclear translocation [87]. MAST3 (S134) was identified as another potential upstream kinase through in silico predictive tools, which exhibits a negative co-regulation with S66 of MRTFB. MAST3 is involved in cytoskeleton organization and intracellular signal transduction [88]. Likewise, CSNK1A1 (T321) was also a potential upstream kinase predicted by Johnson et al. (2023) [45], which showed positive co-regulation with S921 of MRTFB. CSNK1A1 is a critical regulator of the Wnt signaling pathway [89].

### 3.8. Interaction Co-Regulation of Predominant MRTFB Phosphosites Within the Interactome Network

Phosphorylation plays an important role in protein–protein interaction. To analyze this, we combined a list of binary and complex interactors of MRTFB from multiple databases. We then identified seven phosphosites that showed positive co-regulation with the predominant site S66. Similarly, we identified four phosphosites that show positive co-regulation with the site S921 (Table S3C). From the known binary interactors, two phosphosites in the protein MAPK1 show a role in cytoskeleton reorganization, and this was predicted as an upstream kinase of MRTFB by Johnson et al. (2023) [45,90,91,92]. The sites are T185 and Y187 of MAPK1, which also show positive co-regulation with the predominant site S66.

We also identified a set of complex interactors that interact with the predominant sites of MRTFB. At the phosphosite level, these show positive and negative co-regulation with predominant sites of MRTFB (Table S3D). Phosphosite S66 shows positive co-regulation with 172 phosphosites and negative co-regulation with 41 phosphosites. Among the positively co-regulated complex interactors, MAPK1_T185, Y187, and PPP1R12A_S507—and among the negatively co-regulated complex interactors, PXN_Y88—show a role in cytoskeletal reorganization [90,91,92,93,94]. Similarly, phosphosite S921 shows 223 positively and 28 negatively co-regulated phosphosites, which also show complex interaction. Among the positively co-regulated complex interactors, three of them—ABI1_S216, NUSAP1_S240, and KIF2C_S115—show a role in cytoskeletal reorganization [95,96,97,98].

### 3.9. Conserved Phosphorylation Patterns Across the MRTF Family and Species

**Figure 9:**
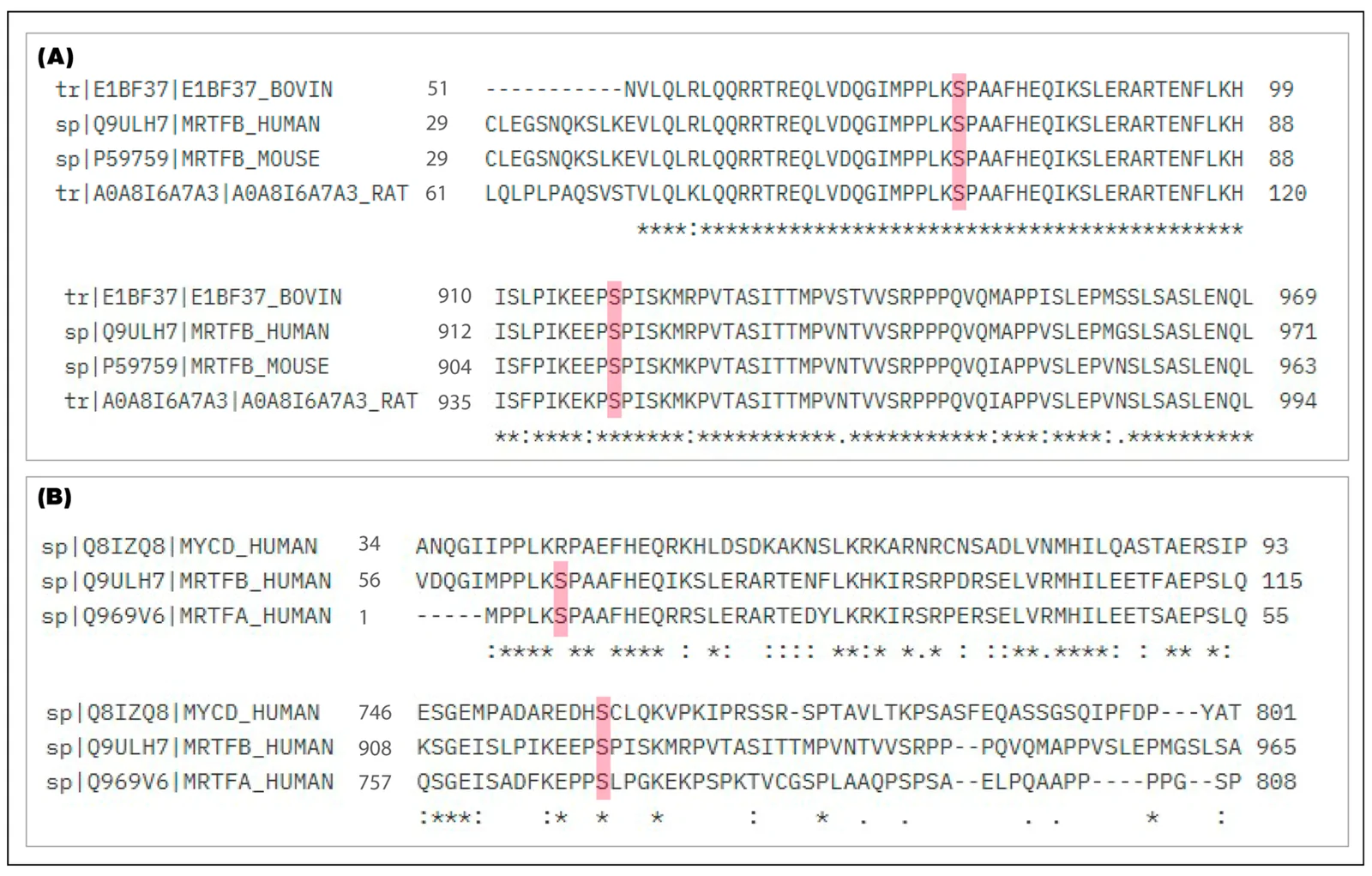
Conservation of MRTFB-predominant phosphosites across species and within the MRTF family of proteins. The predominant phosphosites and the corresponding conserved residues are highlighted in red. (**A**) Conservation of predominant phosphosites S66 and S921 of MRTFB across species (MRTFB_Bovine, MRTFB_Human, MRTFB_Mouse, and MRTFB_Rat). (**B**) Conservation of predominant phosphosites S66 and S921 of MRTFB across the MRTF family (MYOCD_Human, MRTFB_Human, and MRTFA_Human). “*” indicates fully conserved residue (identical), “:” indicates conservative mutation, “.” indicates semi-conservative mutation, “---” indicates gap, “*space*” indicates no significant conservation.

Phosphosite conservation analysis is an effective way to assess functional importance, as highly conserved sites are likely critical for function, since phosphomutations may disrupt protein activity and impede evolutionary selection [99]. The MRTFB protein is evolutionarily conserved between different species. MRTFB shares highly conserved structural domains and functional motifs with other members of the MRTF family. In particular, MRTFA and MRTFB are very closely related proteins and share similar functions in SRF activation [25]. The predominant phosphosites of MRTFB were conserved both within the MRTF family and across vertebrate species, including mouse, rat, and bovine. The N-terminal phosphosite, S66 of human MRTFB, was conserved with S66 of mouse, S98 of rat, and S77 of bovine orthologs. The phosphosite S921 of human MRTFB was also conserved, with S913 in mouse, S944 in rat, and S919 in bovine orthologs (Figure 9A). Furthermore, conservation analysis of predominant phosphosites among the MRTF family showed that S66 of human MRTFB was conserved as S6 in human MRTFA, but it is not conserved in MYOCD. Additionally, the predominant phosphosite residue S921 was conserved within the MRTF family, aligning with S770 of human MRTFA and S759 of human MYOCD (Figure 9B).

### 3.10. Co-Regulated Proteins of MRTFB-Predominant Phosphosites in Actin Cytoskeleton Reorganization Pathway

The positively and negatively co-regulated PsOPs associated with the predominant phosphosites of MRTFB were involved in cytoskeletal reorganization. We identified six PsOPs from positive co-regulation of S66 and two PsOPs from negative co-regulation of S66, which were associated with cytoskeleton reorganization. Similarly, S921 showed nine PsOPs from positive co-regulation data. The negative co-regulation data showed no PsOPs that are associated with cytoskeleton reorganization. These PsOPs, which are positively or negatively co-regulated with the predominant phosphosites of MRTFB that are associated with cytoskeleton reorganization, are mentioned in Table 1.

Additionally, we performed pathway enrichment analysis of the co-regulated proteins of predominant sites S66 and S921. Among the enriched pathways, “regulation of actin cytoskeleton pathway” was found to be associated with MRTFB’s function, with an FDR value of 7.1 × 10^−4^ and a fold enrichment value of 2.7 (Figure S1, Table S4A). Among the co-regulated proteins, the proteins involved in the “regulation of actin cytoskeleton pathway” are adenomatous polyposis coli (APC), breast cancer anti-estrogen resistance protein 1 (BCAR1), dedicator of cytokinesis 1 (DOCK1), mitogen-activated protein kinase 1 (MAPK1), mitogen-activated protein kinase 3 (MAPK3), ezrin (EZR), G protein-coupled receptor kinase-interacting protein 1 (GIT1), Rho GTPase-activating protein 35 (ARHGAP35), IQ motif containing GTPase activating protein 3 (IQGAP3), integrin subunit beta 4 (ITGB4), LIM domain kinase 1 (LIMK1), LIM domain kinase 2 (LIMK2), protein diaphanous homolog 1 (DIAPH1), protein enabled homolog (ENAH), protein phosphatase 1 regulatory subunit 12A (PPP1R12A), protein phosphatase 1 regulatory subunit 12C (PPP1R12C), solute carrier family 9 member A1 (SLC9A1), paxillin (PXN), B-Raf proto-oncogene, serine/threonine kinase (BRAF), Rho guanine nucleotide exchange factor 12 (ARHGEF12), Rho guanine nucleotide exchange factor 1 (ARHGEF1), Son of sevenless homolog 1 (SOS1), slingshot protein phosphatase 1 (SSH1), and slingshot protein phosphatase 2 (SSH2) (Figure S2).

Furthermore, we also performed functional enrichment analysis of co-regulated proteins of predominant sites. The positively co-regulated proteins of phosphosite S66 showed association with regulation of cytoskeleton organization, regulation of supramolecular fiber organization, cytoskeleton organization, and actin cytoskeleton organization (Figure S3, Table S4B). The positively co-regulated proteins of phosphosite S921 were associated with chromatin organization, chromosome organization, regulation of chromosome organization, and regulation of cell cycle process (Figure S4, Table S4C).

## 4. Discussion

This study provides insights into the role of MRTFB in actin binding and nuclear localization, the involvement of proteins co-regulated with the predominant phosphosites in the regulation of the actin cytoskeleton pathway, and the conservation of predominant phosphosites across species and the MRTF family. By analyzing phosphoproteomic datasets, we identified two predominant phosphosites (S66 and S921) with high detection frequency across profiling and differential expression datasets. Specifically, our analysis revealed that these predominant phosphosites of MRTFB are conserved across the MRTF family and distinct species.

Panayiotou et al. (2016) reported that S98 (same as S41) of mouse MRTFA is phosphorylated by ERK upon stimulation and activated Raf/MEK upon TPA stimulation, in a U0126-sensitive fashion. This phosphorylation destabilizes the G-actin/MRTFA complex by reducing the stoichiometry of G-actin binding to RPEL1, thus facilitating nuclear import and nuclear localization signal (NLS) [100]. The S66 site in human MRTFB is also known to dissociate actin binding and to facilitate nuclear translocation, thereby activating SRF genes [6]. The site S913 of mouse MRTFB is phosphorylated by MAPK1 (ERK2). This site also promotes nuclear localization, thereby enhancing SRF-mediated gene transcription [101]. Even though the site S913 of mouse MRTFB (conserved with S921 of human MRTFB) is phosphorylated by MAPK1, corresponding studies on the human ortholog of MRTFB have not yet been reported. Furthermore, the predominant phosphosites S66 and S921 are found to be the most positively co-occurring, with a ratio of 21. Thus, these sites may exhibit potential co-regulation within shared or converging regulatory mechanisms.

SRF activation is regulated by both actin dynamics and kinase signaling pathways, particularly with myocardin-related transcription factors (MRTFs), such as MRTF-B (MKL2). In the resting state, MRTFB is situated in the cytoplasm through its binding with monomeric G-actin. When Rho GTPase stimulates actin polymerization, the G-actin level decreases, resulting in the dissociation of MRTFB from actin and its translocation into the nucleus. Once in the nucleus, MRTFB enables SRF coactivation to drive the expression of genes associated with cytoskeletal organization [18,19]. Single-molecule analyses of SRF dynamics show that stimulation-dependent increases in long-duration SRF chromatin binding correlate with MRTF activation and are modulated by both actin remodeling and MAPK pathways [102]. Through these mechanisms, extracellular signals are integrated with changes in actin dynamics to regulate SRF-dependent gene expression. MRTFB also functions within broader regulatory networks. For instance, MRTF family members are involved in crosstalk with Hippo signaling by binding YAP through a conserved PPXY motif that interacts with the YAP WW domain, recruiting NCoA3 to the TEAD–YAP complex to potentiate target transcription [103]. Conversely, MRTFB activity is negatively regulated through direct physical interaction with SMAD3 and loss of SMAD3 leads to MRTF disinhibition and hyperactivation [104]. Additionally, MRTFB operates in a reciprocal transcriptional repression loop with Bcl6 via the MKL/SRF complex during cellular differentiation processes [105].

The pathway enrichment analysis shows that the “regulation of actin cytoskeleton” pathway has functional correlation with MRTFB. This pathway controls the cytoskeletal structure, cell shape, motility, and gene transcription through MRTF and SRF. The proteins co-regulated with the predominant phosphosites of MRTFB that are involved in this pathway are upstream RhoGEFs (ARHGEF1/12) [19], focal adhesion sensors including PXN, BCAR1, and GIT1 [106,107,108], as well as the transmembrane protein ITGB4 [109] and the cytoplasmic scaffolding protein APC [110]. Additionally, formins (DIAPH1) and ENAH nucleate and elongate actin filaments, while downstream effectors ROCK-LIMK phosphorylate cofilin, inhibiting actin depolymerization. The slingshot phosphatase (SSH1/SSH2) reactivates cofilin to promote filament severing [19]. Furthermore, BRAF is a key regulator of the actin cytoskeleton in the context of cancer [111].

By integrating the known and predicted data of upstream kinases and interactors into the PsOP network, we identified potential upstream kinases and interactors of MRTFB that are involved in cytoskeleton reorganization. The predicted upstream kinases that are positively or negatively co-regulated with the predominant MRTFB sites are MAPK1 T185 and Y187, MAPK3 T207 and T198, MAST3 S134, and CSNK1A1 T321. MAPK1 (mitogen-activated protein kinase 1) is a binary interactor of MRTFB, which is part of a feedback loop. The MAPKs (including MAPK1 and MAPK3) can modulate the actin cytoskeleton, which regulates the localization of MRTFB, while MRTFB acts as a transcriptional co-activator that restructures the actin cytoskeleton in response to signaling activation [112,113,114,115]. MAPKs can also phosphorylate/activate proteins associated with actin cytoskeleton organization [115]. The recent findings (Lubaba et al., 2025) [88] indicate that MAST3 (microtubule-associated serine/threonine-protein kinase 3) is predicted to be an upstream kinase and to be associated with actin cytoskeletal regulation. The CSNK1A1 (Casein Kinase 1 Alpha 1) regulates intermediate filament cytoskeleton organization, similar to MRTFB’s role in actin cytoskeletal reorganization [112,114,116]. Thus, we can hypothesize that MRTFB and its predicted upstream kinases are involved in cytoskeleton organization and are functionally correlated. However, further studies are needed to confirm their roles at specific phosphorylation sites.

## 5. Limitations of the Study

While this study provides a comprehensive analysis of the phosphoregulatory network of MRTFB by integrating publicly available phosphoproteomic datasets, several limitations must be addressed. First, relying on publicly accessible mass spectrometry datasets introduces inherent heterogeneity arising from variations in cell lines, tissue types, enrichment strategies, and analytical platforms across different studies. Although stringent filtering, experimental condition grouping, and confidence cutoffs were applied to isolate high-confidence phosphosites (S66 and S921) and their co-regulated networks, the absence of direct biochemical and cellular validation leaves the functional impacts of these site-specific modifications to be further confirmed. Furthermore, computational modeling identified predicted candidate upstream kinases such as MAPK1, MAPK3, MAST3, and CSNK1A1 of MRTFB; these predicted kinase–substrate relationships require experimental validation. Future experimental investigations utilizing site-directed mutagenesis, targeted in vitro kinase assays, and quantitative nucleocytoplasmic shuttling assays will be essential to establish the precise mechanistic roles of S66 and S921 in regulating MRTFB nuclear translocation, SRF co-activation, and actin cytoskeleton crosstalk.

## 6. Conclusions

In summary, our study provides a comprehensive analysis of MRTFB phosphorylation, establishing its role in cytoskeletal signaling and nuclear localization, identifying key co-regulated proteins in the “regulation of actin cytoskeleton” pathway, while demonstrating the structural conservation of its predominant phosphosites across species and the MRTF family. Here, we focused on the predominant sites S66 and S921, which were frequently detected in several experimental conditions. First, we analyzed the PsOPs that are positively or negatively co-regulated with the predominant MRTFB sites. We also analyzed the co-occurrence pattern of phosphosites in MRTFB and found that the two predominant sites co-occur, suggesting potential co-regulation within related signaling context. Then we performed pathway enrichment analysis and found the “regulation of actin cytoskeleton” enriched pathway has a role in MRTFB’s and its co-regulated proteins’ function.

## Figures and Tables

**Table 1:** PsOPs that are co-regulated with predominant phosphosites of MRTFB, which are also involved in cytoskeletal reorganization at the phosphosite level.

MRTFB Phosphosites	Co-Regulated Protein Phosphosites	Cytoskeletal Reorganization Reference PMID	Co-Regulation Type	Co-Regulation Frequency
MRTFB_S66	MAPK1_Y187	19046339, 18045996, 17562849	Positive	13.66
MRTFB_S66	MAPK1_T185	19046339, 18045996, 17562849	Positive	12.66
MRTFB_S66	MISP_S400	34023777	Positive	11.5
MRTFB_S66	MAPRE1_S155	30250248, 24930764	Positive	11
MRTFB_S66	PPP1R12A_S507	37064875	Positive	8.5
MRTFB_S66	MISP_S395	34023777	Positive	6.5
MRTFB_S66	FERMT2_S159	32104508, 25646088	Negative	8
MRTFB_S66	PXN_Y88	35297710	Negative	7
MRTFB_S921	ABI1_S216	21900237, 21419341	Positive	10
MRTFB_S921	LCP1_S5	33449151, 23001012, 20169155	Positive	10
MRTFB_S921	FHOD1_S498	31794718	Positive	9.66
MRTFB_S921	KPNA2_S62	33526712	Positive	7.25
MRTFB_S921	NUMA1_T2055	23921553	Positive	7
MRTFB_S921	NUSAP1_S240	38117947	Positive	7
MRTFB_S921	GPHN_S270	33796945	Positive	7
MRTFB_S921	KIF2C_S115	14960279	Positive	7
MRTFB_S921	EPHA2_S897	25908849	Positive	6.66

## Data Availability

The original contributions presented in this study are included in the article/Supplementary Material. Further inquiries can be directed to the corresponding author(s).

## Supplementary Materials

The following supporting information can be downloaded at https://www.mdpi.com/article/10.3390/cimb48090910/s1.
