# Supplementary material for: Systematic Analysis of MRTFB Phosphoproteomic Datasets Reveals Co-Regulated Kinase Networks and Co-Occurring Phosphosites Associated with the Actin Cytoskeleton Pathway
Source: Curr Issues Mol Biol. 2026 Sep 5;48(9):910. doi: 10.3390/cimb48090910 (PMC13605766; doi:10.3390/cimb48090910)
Supplement: Supplementary file 1 [file cimb-48-00910-s001.zip › Supplementary Figures.pdf]

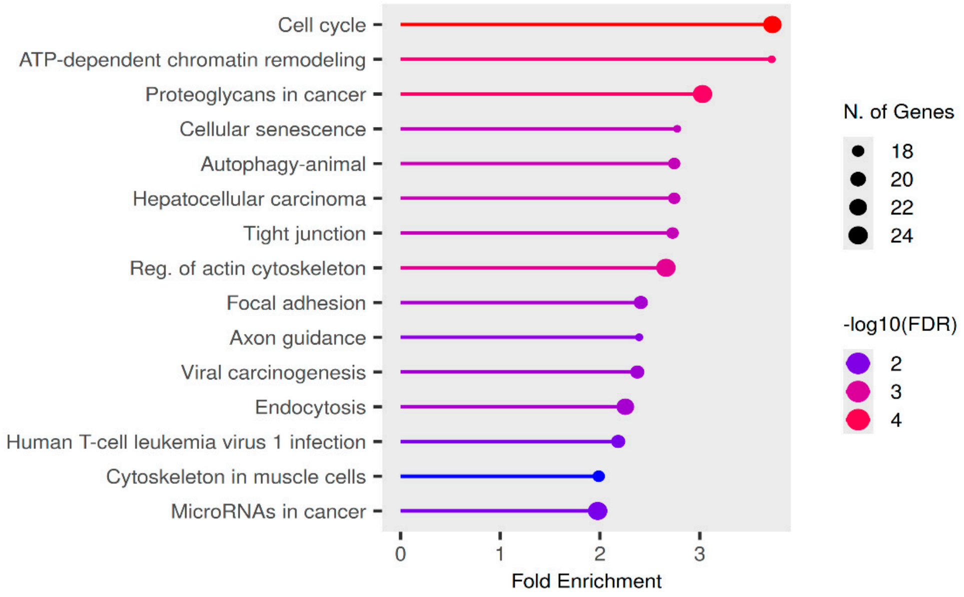

Figure S1: Pathways enriched with the co-regulated proteins of MRTFB.

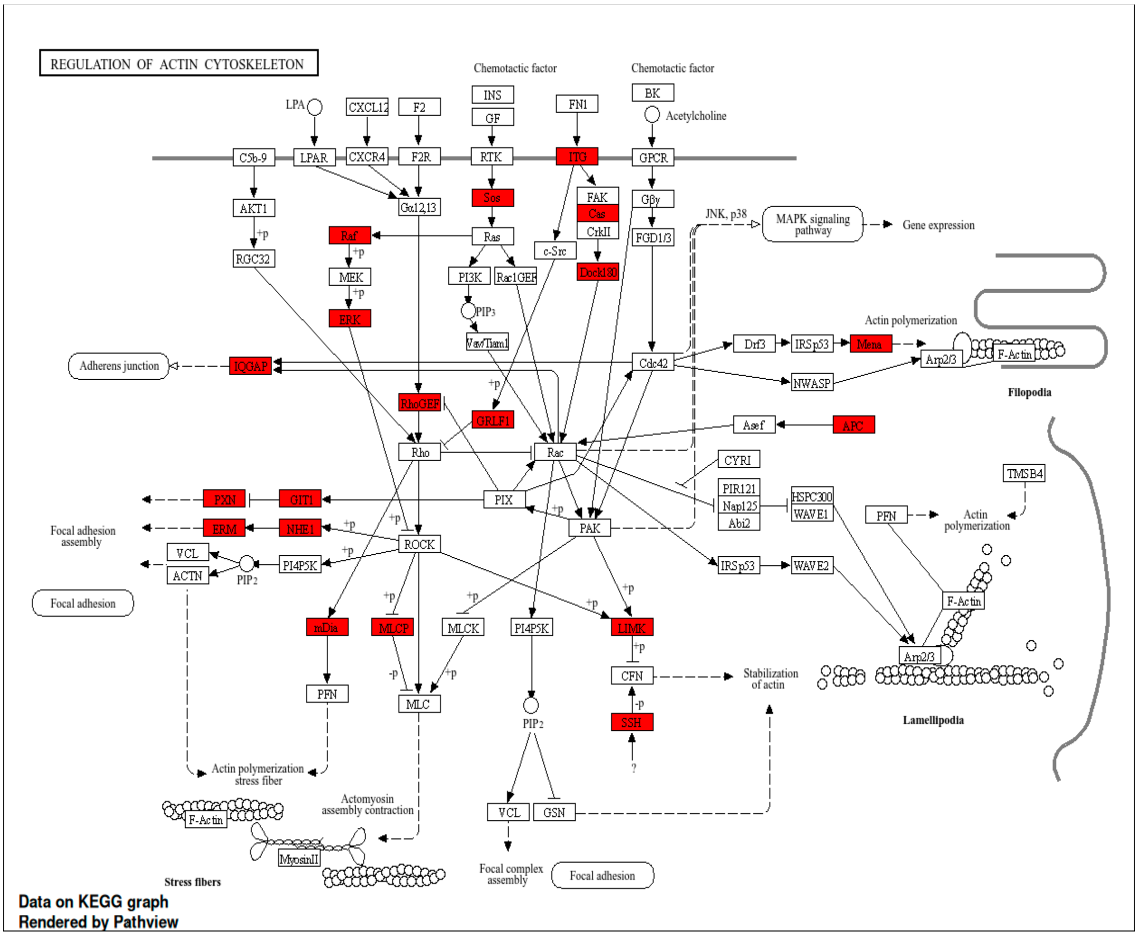

Figure S2: Pathway enrichment of co-regulated proteins with the predominant phosphosites of MRTFB. Co-regulated proteins enriched in the ‘Regulation of Actin Cytoskeleton' pathway are highlighted in red.

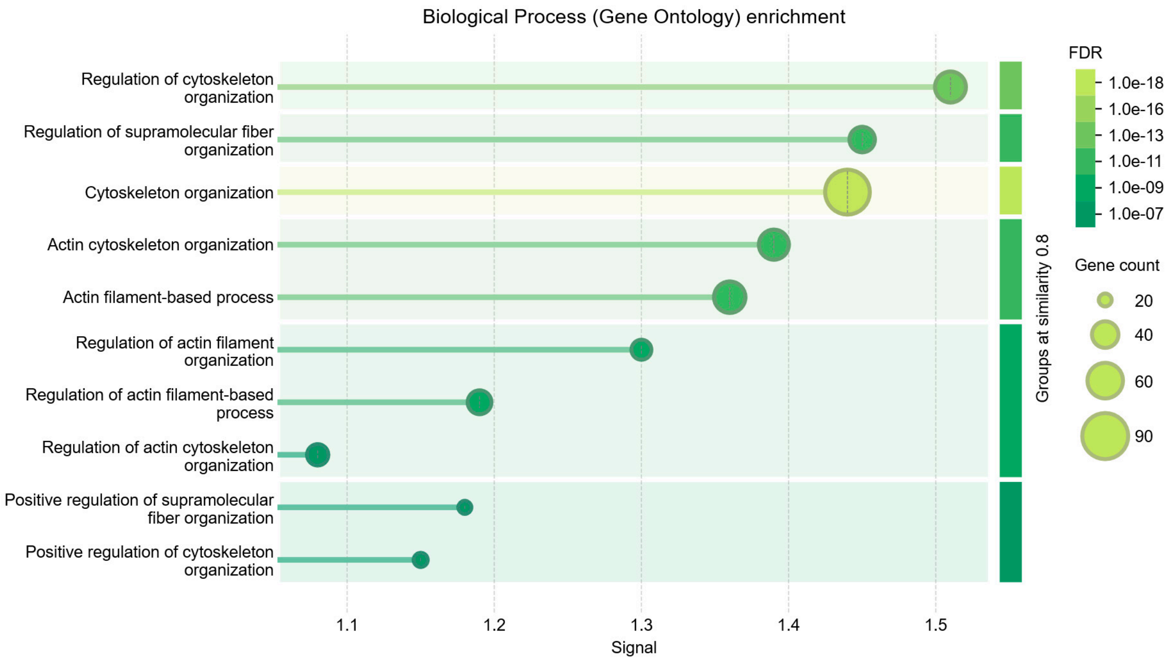

Figure S3: Functional enrichment of positively co-regulated proteins of predominant phosphosite S66.

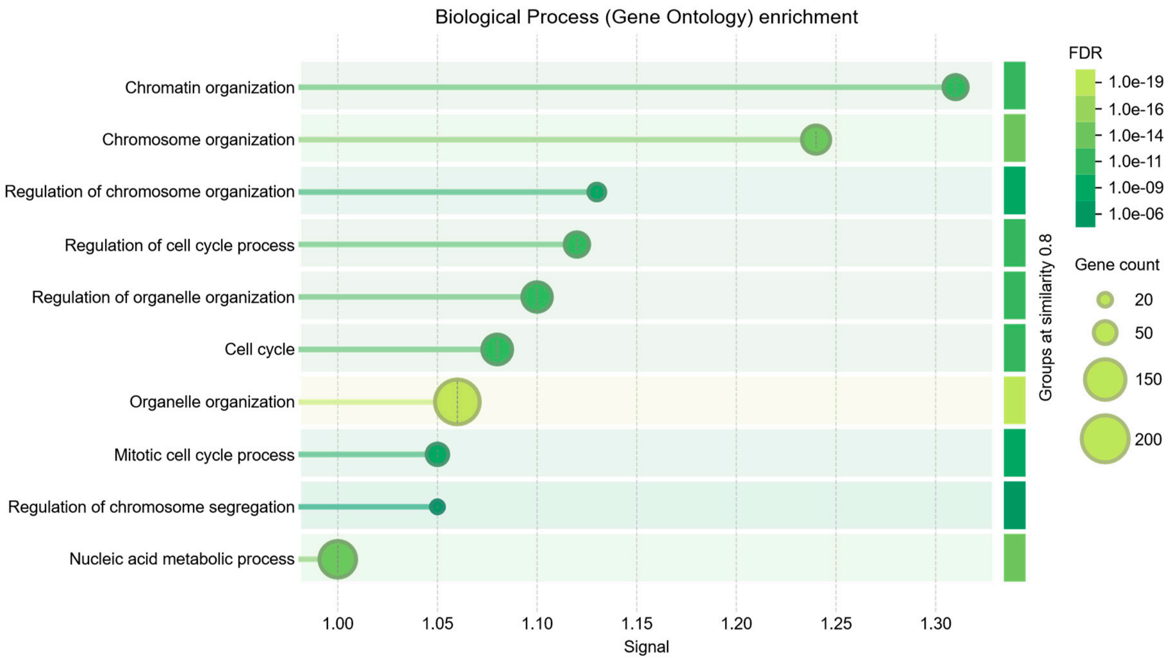

Figure S4: Functional enrichment of positively co-regulated proteins of predominant phosphosite S921.
